# Uncontrolled hypertension among patients managed in primary healthcare facilities in Kinshasa, Democratic Republic of the Congo

**DOI:** 10.5830/CVJA-2016-036

**Published:** 2016

**Authors:** TM Kika, EV Kintoki, JR M’Buyamba-Kabangu, FB Lepira, JR Makulo, EK Sumaili, PK Kayembe

**Affiliations:** Division of Cardiology, University of Kinshasa Hospital, Kinshasa, Democratic Republic of the Congo; Division of Cardiology, University of Kinshasa Hospital, Kinshasa, Democratic Republic of the Congo; Division of Cardiology, University of Kinshasa Hospital, Kinshasa, Democratic Republic of the Congo; Division of Nephrology and Hypertension, Kinshasa School of Public Health, Kinshasa, Democratic Republic of the Congo; Division of Nephrology and Hypertension, Kinshasa School of Public Health, Kinshasa, Democratic Republic of the Congo; Division of Nephrology and Hypertension, Kinshasa School of Public Health, Kinshasa, Democratic Republic of the Congo; Department of Internal Medicine, University of Kinshasa, Kinshasa, Democratic Republic of the Congo

**Keywords:** uncontrolled hypertension, risk factors, primary care, black Africans

## Abstract

**Background:**

Uncontrolled hypertension remains an important issue in daily clinical practice worldwide. Although the majority of patients are treated in primary care, most of the data on blood pressure control originate from populationbased studies or secondary healthcare.

**Objective:**

The aim of this study was to evaluate the frequency of uncontrolled hypertension and associated risk factors among hypertensive patients followed at primary care facilities in Kinshasa, the capital city of Democratic Republic of the Congo.

**Methods:**

A sample of 298 hypertensive patients seen at primary healthcare facilities, 90 men and 208 women, aged ≥ 18 years, were consecutively included in this cross-sectional study. The majority (66%) was receiving monotherapy, and diuretics (43%) were the most used drugs. According to 2007 European Society of Hypertension/European Society of Cardiology hypertension guidelines, uncontrolled hypertension was defined as blood pressure ≥ 140/90 or ≥ 130/80 mmHg (diabetes or chronic kidney disease). Logistic regression analysis was used to identify the determinants of uncontrolled hypertension.

**Results:**

Uncontrolled hypertension was observed in 231 patients (77.5%), 72 men and 159 women. Uncontrolled systolic blood pressure (SBP) was more frequent than uncontrolled diastolic blood pressure (DBP) and increased significantly with advancing age (p = 0.002). The proportion of uncontrolled SBP and DBP was significantly higher in patients with renal failure (p = 0.01) and those with high (p = 0.03) to very high (p = 0.02) absolute cardiovascular risk. The metabolic syndrome (OR 2.40; 95% CI 1.01–5.74; p = 0.04) emerged as the main risk factor associated with uncontrolled hypertension.

**Conclusion:**

Uncontrolled hypertension was common in this case series and was associated with factors related to lifestyle and diet, which interact with blood pressure control.

## Background

Hypertension is the most prevalent treatable cause of cardiovascular (CVD) and chronic kidney disease (CKD).^1^ Controlling hypertension leads to significant reduction in the prevalence and incidence of target-organ damage (TOD) and mortality from CVD.^1^ Despite the availability of effective antihypertensive treatments and guideline recommendations on the management of high blood pressure, hypertension remains one of the most poorly controlled risk factors in patients with and without CVD.^1^ This highlights the need to consider lifestyle and diet as well as tolerance and adherence to treatment with several antihypertensive drugs.^1^ Controlling hypertension often requires the use of several antihypertensive agents, especially in elderly patients or those with stroke or diabetes.^1^

Patients with hypertension and one or more co-morbidities increasingly form a significant part of the primary care practitioner’s case load.^2^ Insufficient blood pressure control remains an important issue in daily clinical practice worldwide.^3^ However, most of the international data on blood pressurecontrol studies originate from national surveys, populationbased studies or secondary healthcare, despite the fact that the majority of patients are treated in a primary care (PC) setting.^3^ Therefore, data on hypertension treatment and control from PC are awaited.

In Democratic Republic of the Congo (DRC), despite the ever-growing prevalence of hypertension, data on uncontrolled hypertension are scarce and rely upon few population-based studies.^4^-^6^ Therefore, the objective of this study was to evaluate the frequency of uncontrolled hypertension and associated risk factors among hypertensive patients followed at primary care facilities in Kinshasa, the capital city, with an estimated population of 10 million.

## Methods

From 30 April to 24 August 2012, all consecutively appearing patients with known hypertension and regularly followed at healthcare centres of the Roman Catholic Church Network (BDOM) were asked to participate in this cross-sectional study. Inclusion criteria were: age ≥ 18 years, being on antihypertensive treatment for at least three months, and giving a written informed consent to participate in the study.

Data were obtained using a standard questionnaire, which collected information on age, gender, education level, duration of hypertension, the number and class of drugs taken for hypertension or other conditions, compliance with antihypertensive drugs, family history of hypertension (FH-HT), diabetes (FH-DM) or cardiovascular disease (CVD), smoking and alcohol use, and physical activity.

A physical examination was performed on each patient to measure height, weight, waist circumference (WC), blood pressure and pulse rate. Height and weight were measured with reference to recommended procedures. Body mass index (BMI) was calculated as weight (kg)/height^2^ (m). Overweight and obesity were defined as BMI > 25 and > 30 kg/m^2^.^7^ Waist circumference (WC) was taken to the nearest 1 cm, using a tape measure. Central obesity was defined as WC > 94 cm in men and > 80 cm in women.^8^

Seated blood pressure (BP) was measured using an electronic device, OMRON M3 Intellisense (OMRON Health, Vietnam), on the left arm at the level of the heart after five minutes’ rest. BP was measured three times and the mean of the last two readings was used for analysis. Pulse pressure (PP) was calculated as systolic (SBP) minus diastolic blood pressure (DBP). Mean arterial pressure (MAP) was DBP + PP divided by 3.

While on their usual diet, a venous blood sample was taken from an antecubital vein for the determination of levels of serum uric acid, cholesterol and its sub-fractions, and triglycerides using enzymatic methods (Biomérieux France). Low-density lipoprotein cholesterol (LDL-C) was calculated using the Friedewald formula.^9^

For estimated glomerular filtration rate (eGFR) determinations, the abbreviated equation from the MDRD study was used.^10^ We calibrated the creatinine results measured using the Jaffe method against a traceable isotope dilution mass spectrometry (IDMS) enzymatic method (creatinine +, Roche enzymatic diagnostics) as described elsewhere.^11^ Recalibrated serum creatinine values were thereafter computed for each participant and the new MDRD study equation was used for estimation of eGFR as 175 × [serum creatinine level (mg/dl)] – 1.154 × [age (years)] – 0.203. For women and for blacks (all patients in our study), the product of this equation was multiplied by a correction factor of 0.742 and 1.21, respectively. All analyses were performed at the National Laboratory of the National AIDS Control Program.

Capillary blood glucose was determined using Accu-chek Compact plus glucometer (Roche Diagnostica, Manheim, Germany) with the glucose oxidase method. Reactive Dipstick Condor Teco (Condor Teco Medical Technology Co, China) was used to determine semi-quantitative proteinuria. A resting electrocardiogram (ECG) was performed for each patient and the Sokolow index was calculated. The 2007 European Society of Hypertension/European Society of Cardiology (ESH/ESC) guidelines^12^ were used to evaluate global cardiovascular (CV) risk in the study population.

Subjects were classified as having: controlled SBP and DBP if current antihypertensive treatment was accompanied by clinic SBP < 140 mmHg and clinic DBP < 90 mmHg; uncontrolled SBP only if SBP was ≥ 140 mmHg and DBP < 90 mmHg; uncontrolled DBP only if DBP was ≥ 90 mmHg and SBP < 140 mmHg; uncontrolled SBP and DBP if SBP and DBP were ≥ 140 mmHg and ≥ 90 mmHg, respectively.^12^

The metabolic syndrome was defined according to International Diabetes Federation criteria.8 Diabetes was defined as blood glucose level ≥ 126 mg/dl (6.99 mmol/l) or current use of antidiabetic drugs.^13^

Excessive alcohol intake was defined by regular intake of two or more glasses per day of beer or equivalent for at least one year, knowing that one glass of beer contains 10 g of alcohol.^14^ Smoking was defined as regular consumption of at least one cigarette per day for more than five years or having stopped smoking for less than five years.^15^

Physical activity in leisure time was categorised as active for subjects who exercised for at least four hours per week, and inactive or sedentary for all the others.^16^ Compliance with therapy was defined as self-reported regular intake of antihypertensive drugs.

ECG-determined left ventricular hypertrophy (ECG-LVH) was defined as a Sokolow index > 35 cm.^17^ According to K/DOQI guidelines,18 chronic kidney disease (CKD) and renal failure (RF) were defined as eGFR < 90 and < 60 ml/min/1.73 m^2^, respectively. According to the 2007 ESH/ESC guidelines,12 moderate, high and very high absolute CV risk were defined as 10–20, 20–30 and ≥ 30% probability of a CV event in the next 10 years, respectively. Proteinuria was defined as dipstick proteinuria ≥ 1+.^19^

The study was conducted in accordance with the principles of the 18th World Assembly (Helsinki, 1964). The study protocol was submitted to the ethics committee of Kinshasa School of Public Health of the University of Kinshasa and received clearance under the number ESP/CE/024/2012.

## Statistical analysis

Data are expressed as mean ± standard deviation (SD) or relative frequency (%). Chi-squared and Student’s t-tests were used to compare categorical and continuous variables, respectively. Skewed continuous variables were compared using the non-parametric Mann–Whitney test. Stepwise logistic regression analysis was used to identify correlates of uncontrolled hypertension; odds ratio (OR) and confidence interval (CI) were obtained for each independent variable. To remain in the model a factor had to reach a p-value ≤ 0.05. All statistical analyses were performed with SPSS version 20 for Windows at the Division of Epidemiology and Biostatistics of Kinshasa School of Public Health, University of Kinshasa.

## Results

A total of 298 hypertensive patients, 208 women and 90 men, were recruited in this study. Clinical characteristics of the study population as a whole are given in Table 1. Their mean age was 64 ± 10 years; they had on average a BMI of 26 ± 5 kg/m^2^, a WC of 90 ± 11 cm, a SBP of 151 ± 24 mmHg and a DBP of 87 ± 14 mmHg.

**Table 1:** Clinical characteristics of the study population as a whole and by blood pressure control status

**	*Whole group*	*Controlled HT*	*Uncontrolled HT*	**
*Variable*	*(n = 298)*	*(n = 67)*	*(n = 231)*	*p-value*
Age, years	64 ± 10	64 ± 10	64 ± 10	
Gender, %				
Males	30	27	31	0.548
Females	70	73	69	
DHT, %				0.179
< 1 year	15	16	14	
1–4 years	29	39	26	
5–9 years	23	19	24	
≥ 10 years	33	26	35	
FH-HT, %	50	49	50	0.851
FH-DM, %	31	22	34	0.166
BMI, kg/m^2^	26 ± 5	26 ± 5	25 ± 5	0.238
WC, cm	90 ± 11	90 ± 11	90 ± 11	0.953
SBP, mmHg	151 ± 24	122 ± 10	160 ± 20	0.001
DBP, mmHg	87 ± 14	75 ± 7	91 ± 13	0.001
MBP, mmHg	109 ± 16	91 ± 7	114 ± 13	0.001
PP, mmHg	64 ± 19	47 ± 9	69 ± 18	0.001
Pulse rate, bpm	76 ± 13	77 ± 12	77 ± 13	0.493
AntiHT regimen, %				
1 drug	66	75	66	0.171
≥ 2 drugs	34	25	34	
Non-drug compliance, %	42	42	42	0.050

Data are expressed as mean ± standard deviation (SD) or relative frequency (%). DHT, duration of hypertension; FH-HT, family history of hypertension; FH-DM, family history of diabetes mellitus; BMI, body mass index; WC, waist circumference; SBP, systolic blood pressure; DBP, diastolic blood pressure; MBP, mean blood pressure; PP, pulse pressure; bpm, beats per minute; AntiHT, antihypertensive.

A family history of hypertension or diabetes was present in 50 and 31% of patients, respectively. In 67% of patients, the duration of hypertension was less than 10 years. The majority of patients (66%) were receiving monotherapy, most with diuretics (43%) (Table 2). Of the 34% of patients on combined therapy, a notable proportion (17%) was receiving a fixed combination of an angiotensin converting enzyme and a thiazide diuretic. With regard to non-antihypertensive drugs, 29, 9 and 6% of patients were on antidiabetic, non-steroidal anti-inflammatory and antiplatelet drugs, respectively. A sizeable proportion of treated patients (42%) self-reported non-compliance with antihypertensive therapy.

**Table 2:** Antihypertensive and non-antihypertensive drugs in the study population

*Antihypertensive drugs*	*(n = 298)*	*Non-antihypertensive drugs*	*(n = 298)*
Monotherapy, %	66	Lipid-lowering drugs, %	0.3
Diuretic, %	43	Uric acid-lowering drugs, %	0
CCB, %	11	Antiplatelet drugs, %	6
ACEI, %	11	NSAIDs, %	9
CAA, %	1	Antidiabetic drugs,	29
Combined therapy, %	34		
Diuretic + ACEI, %	17		
Diuretic + CCB, %	9		
ACEI + CCB, %	4		
Others, %	4		

Data are expressed as relative frequency (in percent).

CCB, calcium channel blocker; ACEI, angiotensin converting enzyme inhibitor; CAA, central-acting agents; NSAID, non-steroidal anti-inflammatory drugs.

**Fig. 1.**
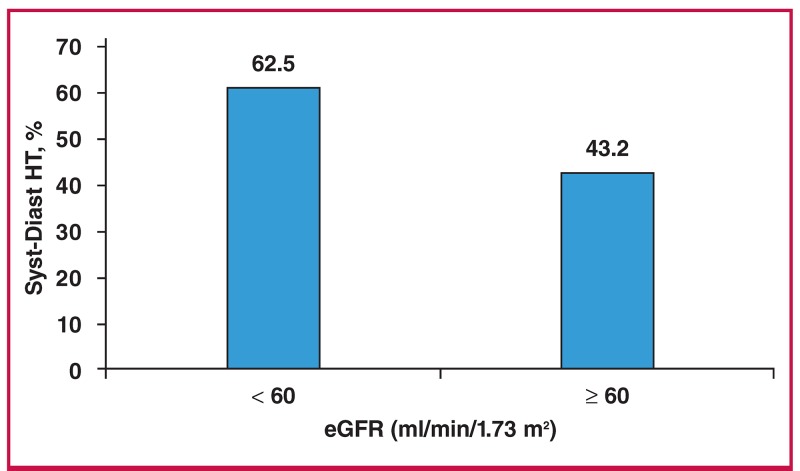
Frequency of uncontrolled systolic and diastolic hypertension (Syst-Diast HT) by renal function status (n = 231).

**Fig. 2.**
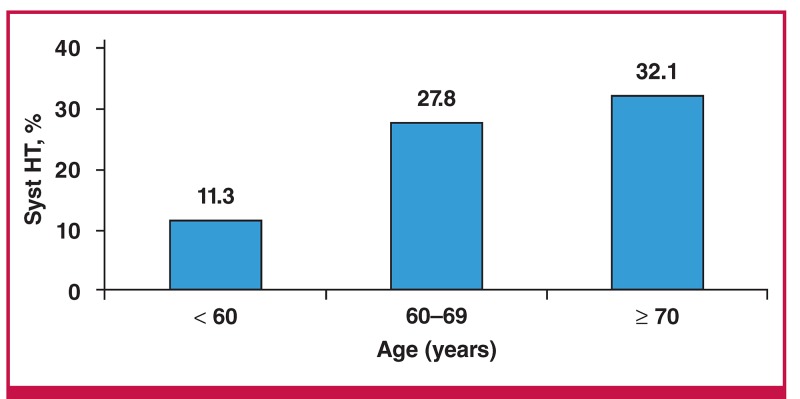
Frequency of uncontrolled systolic hypertension (Syst HT) by age category (n = 231).

Uncontrolled hypertension was observed in 231 patients (77.5%), 72 men and 159 women, of whom 43, 24 and 5% had uncontrolled SBP and DBP, isolated uncontrolled SBP and isolated uncontrolled DBP, respectively (Table 1). The frequency of uncontrolled SBP and DBP was significantly higher in patients with reduced eGFR (62.5 vs 43.2%; p = 0.01) in comparison with those with relatively normal renal function (Fig. 1). Uncontrolled SBP increased significantly (p = 0.002) with advancing age (Fig. 2).

Compared to patients with controlled hypertension (Table 3), those with uncontrolled BP had significantly higher levels of blood glucose (119 ± 14 vs 104 ± 27 mg/dl; p = 0.011); there were also higher numbers of subjects with diabetes (42 vs 22%; p = 0.003), lower high-density lipoprotein cholesterol levels (HDL-C) (24 vs 13%; p = 0.034), and moderate (45 vs 18%; p = 0.006) and high to very high (38 vs 13%; p = 0.02) global CV risk (Table 4). In patients with uncontrolled hypertension, a higher proportion was not compliant with the antihypertensive therapy; but the difference did not reach the level of statistical significance (Table 1).

**Table 3:** Biological characteristics of the study population as a whole and by blood pressure control status

**	**	*Whole group*	*Controlled HT*	*Uncontrolled HT*	**
*Variable*	*n*	*(n = 298)*	*(n = 67)*	*(n = 231)*	*p-value*
Blood glucose, mg/dl	237	115 ± 53	104 ± 27	119 ± 59	0.011
(mmol/l)		6.38 ± 2.94	5.77 ± 1.50	6.60 ± 3.27	
Lipids	259				
TC, mg/dl		220 ± 58	225 ± 60	219 ± 57	0.548
(mmol/l)		5.7 ± 1.5	5.83 ± 1.55	5.67 ± 1.48	0.462
LDL-C, mg/dl		135 ± 55	139 ± 57	134 ± 55	0.537
(mmol/l)		3.50 ± 1.42	3.60 ± 1.48	3.47 ± 1.42	
HDL-C, mg/dl		63 ± 18	62 ± 19	63 ± 18	0.530
(mmol/l)		1.63 ± 0.47	1.61 ± 0.49	1.63 ± 0.47	
TG, mg/dl		111 ± 51	118 ± 62	109 ± 47	0.261
(mmol/l)		1.25 ± 0.58	1.33 ± 0.70	1.23 ± 0.53	
Creatinine, mg/dl	255	1.04 ± 0.56	0.95 ± 0.27	1.07 ± 0.62	0.133
(μmol/l)		91.94 ± 9.50	83.98 ± 23.87	94.59 ± 54.81	
eGFR, ml/min/0.73 m²	255	82 ± 31	86 ± 28	81 ± 32	0.319
Uric acid, mg/dl	259	6.38 ± 2.50	6.60 ± 2.40	6.30 ± 2.50	0.488

Data are expressed as mean ± standard deviation (SD) or relative frequency (%).

TC, total cholesterol ; LDL-C, low-density lipoprotein cholesterol; HDL-C, highdensity lipoprotein cholesterol; TG, triglycerides; eGFR, estimated glomerular filtration rate.

**Table 4:** Cardiovascular risk factors among the study population as a whole and by blood pressure control status

**	**	*Whole group*	*Controlled HT*	*Uncontrolled HT*	**
*Variable*	*n*	*(n = 298)*	*(n = 67)*	*(n = 231)*	*p-value*
Age, %	298	86	87	86	0.989
Smoking, %	298	3	2	3	0.348
Alcohol, %	298	17	18	17	0.462
Overweight, %	298	35	31	36	0.228
Obesity, %	298	17	16	22	0.228
Central obesity, %	298	66	66	66	1.000
Diabetes, %	298	37	22	42	0.003
Hypercholesterolaemia, %	259	17	61	51	0.229
Low HDL-C, %	259	16	13	24	0.034
Hypertriglyceridaemia, %	259	18	18	15	0.700
Hyperuricaemia, %	259	33	33	36	0.210
Dipstick proteinuria, %	227	16	18	12	0.396
Renal failure, %	255	30	12	21	0.103
ECG-LVH, %	164	21	16	14	0.843
MetS, %	298	10	8	15	0.105
Global CV risk, %	298				
Low		31	69	19	0.0001
Moderate		39	18	45	0.0006
High/very high		30	13	38	0.03

Data are expressed as mean ± standard deviation (SD) or relative frequency (%).

HDL-C, high-density lipoprotein cholesterol; ECG-LVH, electrocardiographically determined left ventricular hypertrophy; MetS, metabolic syndrome; CV, cardiovascular risk.

In multivariate analysis, the metabolic syndrome (MetS) and non-compliance with antihypertensive therapy emerged as the main risk factors for uncontrolled hypertension (Table 5). Compared to patients without the MetS, those bearing this metabolic abnormality had a 2.4-fold greater risk (OR 2.4; 95% CI 1.008–5.735; p = 0.04) of having uncontrolled hypertension. Patients not compliant with antihypertensive therapy had a 2.14- fold greater risk (OR 2.14; 95% CI 0.986–4.236; p = 0.05) of uncontrolled hypertension in comparison with those compliant with therapy; but the difference was not statistically significant

**Table 5:** Multivariate independent determinants associated with uncontrolled hypertension

*Variable*	*B*	*SE*	*OR (95% CI)*	*p-value*
Constant	–1.901	0.924	–	–
MetS+ vs MetS–	0.877	0.444	2.40 (1.008–5.735)	0.04

Non-compliance vs compliance

B, regression coefficient; SE, standard error; OR, odds ratio; MetS, metabolic syndrome.

## Discussion

The main findings of this cross-sectional study were as follows: first, uncontrolled hypertension with mainly uncontrolled SBP was frequent in these case series; second, the frequency of uncontrolled hypertension and that of uncontrolled SBP increased with reduced eGFR and advancing age, respectively; third, the MetS emerged as the main risk factor for uncontrolled hypertension.

In this study, 77.5% of patients had uncontrolled hypertension. This observation agrees with previous reports highlighting the fact that in most countries, less than 30% of patients achieve BP goals,^20^ and therapy with a single antihypertensive agent fails to achieve BP goals in up to 75% of patients.^21^

The frequency of uncontrolled hypertension observed in the present study was somewhat higher than that reported in primary care settings by Rayner et al. (60.2%)^22^ and Dennison et al. (64% in the public sector and 49% in the private sector)^23^ in South Africa and by Onwemu et al. (29.4%)^24^ in Nigeria. It was also higher than that observed at tertiary care level by Yaméogo et al. (54.2%)^25^ in Burkina Faso, by Kramoh et al. (56.3%) in Ivory Coast,^26^ and by Ayodele et al. (68.6%)^27^ and Sani et al. (67%)^28^ in Nigeria.

Our clinically generated frequency of uncontrolled hypertension was quite similar to the 76.4% reported by Dzudie et al.^29^ in Cameroon but lower than the 97, 94 and 86.4% previously reported by M’Buyamba-Kabangu et al., Sumaili et al. and Katchunga et al. in the general population of Kinshasa and south-eastern part of Democratic Republic of Congo, respectively.^4^-^6^ Higher frequencies of uncontrolled hypertension ranging from 82.2 to 97.4% were also reported by Hendricks et al. from Namibia to Kenya in a community-based crosssectional study.^30^

Apart from differences in methodology applied and population characteristics studied, the higher frequency of uncontrolled hypertension in sub-Saharan Africa appears to be multifactorial and is determined by patients, care providers and healthcare systems.^31^ Among the factors related to patients, non-compliance with diet and antihypertensive therapy has been reported to be an important determinant of uncontrolled BP.^32^

Non-compliance with antihypertensive therapy emerged in our study as the second risk factor associated with uncontrolled hypertension but the difference was not statistically significant. In many studies, non-compliance with antihypertensive therapy was responsible for two-thirds of the cases of uncontrolled hypertension.^33^,^34^ Krousel-Woods et al.33 found that non-compliance was associated with a nearly two-fold greater risk (OR 1.68; 95% CI 1.01–2.88) of uncontrolled BP. Yaméogo et al.25 in Burkina Faso found that non-compliance with both diet and antihypertensive therapy was associated with an eightfold (OR 8.40; 95% CI 1.11–4.17; p = 0.04) and nearly three-fold greater risk of uncontrolled hypertension, respectively.

With regard to the care provider, clinical therapeutic inertia has been reported to be a major contributor to uncontrolled hypertension.^35^ Although patients with a high to very high CV risk level need more than two antihypertensive drugs to reach the BP goal,^8^ the majority of patients in our study were still on monotherapy, indicating clinical therapeutic inertia. The association of high to very high residual global CV risk has been reported by Yaméogo et al.^25^ in Burkina Faso and Kramoh et al.26 in Ivory Coast, using the Framingham CV risk score and 2007 ESH/ESC guidelines, respectively. In addition, Bohen et al.,^35^ using a cohort of hypertensive diabetics, found that non-intensification of therapy is frequent in this category of patients and is responsible for uncontrolled BP and glycaemia.

Uncontrolled SBP is more frequent and its frequency increases with advancing age, especially after 60 years. A greater frequency of uncontrolled SBP has been reported by Yaméogo et al. in Burkina Faso and Ayodele et al. in Nigeria.^25^,^27^ Significant reduction in systemic arterial elasticity is common with advancing age. This decrease in elasticity results in higher systolic pressures, as large vessels become less able to reduce the pressure generated by the left ventricle by means of distension. On the other hand, while increases in peripheral resistance will cause elevation in diastolic pressure, the loss of large vessel elasticity does the opposite. Therefore, with increasing age, the counteracting forces may keep the diastolic pressure normal, while in the background, there is increasing systolic pressure.^20^,^25^,^26^

In our study, the frequency of uncontrolled SBP and DBP increased with reduced eGFR. Schmitt et al.^36^ reported in a study of 7 227 chronic kidney disease (CKD) patients receiving at least one antihypertensive drug, that only 35% of them had controlled blood pressure. They suggested non-compliance with therapy as the main determinant of uncontrolled hypertension in these CKD patients. Indeed, 33% of patients with CKD were not compliant with therapy and the frequency of non-compliance increased with decreased eGFR.^36^

In univariate analysis, diabetes and low HDL-C levels were significantly associated with uncontrolled hypertension, whereas in multivariate analysis, the MetS and self-reported non-compliance emerged as the main predictors of risk for uncontrolled hypertension; however, the differences observed in non-compliance did not reach the level of statistical significance.

Poor adherence to therapeutic plans and non-compliance with antihypertensive therapy have been reported to be perhaps the most important factors responsible for poor BP control.^37^ In most cases, poverty has been adduced to be responsible for non-compliance, especially in sub-Saharan Africa.^37^ Health education and patient counselling, along with availability of free drugs could help improve adherence to antihypertensive drug therapy.^37^

Previous reports have associated the MetS with an increased risk of uncontrolled hypertension.^38^,^39^ Central obesity via secreted adipocytokines, mainly adiponectin and leptin, appears to be the link between the MetS and uncontrolled hypertension.^38^ Adiponectin, besides its effects on insulin sensitivity, may act directly on the vasculature; indeed, hypo-adiponectinaemia was found to be associated with impaired endothelium-dependent dilation in humans.^38^ Furthermore, leptin has also been reported to increase sympathetic tone and therefore the renin– angiotensin system, with subsequent increase in vascular tone and remodelling.^38^

Several potential limitations of the study need to be underscored. The cross-sectional design of the study did not allow us to establish clear evidence of a causal relationship between the variables of interest. The study sample size was not large enough to be empowered to detect additional associations. The frequency of uncontrolled hypertension could have been overestimated by the lack of inclusion of home blood pressure monitoring while defining BP control.^40^ The non-quantitative evaluation of compliance with antihypertensive therapy may have caused underestimation of this important determinant of BP control; the same could be true for socio-economic status and compliance with diet, especially salt intake. The use of a clinically based sample may limit generalisation of the conclusions of this study to the entire hypertensive population because of bias in referral of patients to the source of care.

## Conclusion

Uncontrolled hypertension was frequent in the present case series and was associated with factors related to lifestyle and diet, which interact with blood pressure control.
